# Supplementary material for: The structure of the complete extracellular bacterial flagellum reveals the mechanism of flagellin incorporation
Source: Nat Microbiol. 2025 Jul 1;10(7):1741–57. doi: 10.1038/s41564-025-02037-0 (PMC12221982; doi:10.1038/s41564-025-02037-0)
Supplement: Supplementary file 1 — Supplementary Table 1 and Supplementary Discussion. [file 41564_2025_2037_MOESM1_ESM.pdf]

nature microbiology

Supplementary information

https://doi.org/10.1038/s41564-025-02037-0

The structure of the complete extracellular bacterial flagellum reveals the mechanism of flagellin incorporation

In the format provided by the authors and unedited

Cryo-EM data collection, refinement and validation statistics

	#1 FliD cap (EMD-51486) (PDB 9GNZ)	#2 HFJ (EMD-51493) (PDB 9GO6)	#3 Cap-HFJ (EMD-51557) (PDB 9GSX)	#4 Filament tip (EMD-51555)
**Data collection and processing**				
Magnification	81,000			
Voltage (kV)	300		300	300
Electron exposure (e–/Å^2)	43		50	
Defocus range (μm)	-0.9 to -1.7		-1.5 to -3.0	
Pixel size (Å)	1.1		2.2	
Symmetry imposed	C1	C1	C1	C1
Initial particle images (no.)	527,490	5,785,833	79,106	
Final particle images (no.)	15,225	65,561	15,077	
Map resolution (Å)	0.143	0.143	0.143	
FSC threshold				
Map resolution (Å)	3.7	2.9	6.2	
**Refinement**				
Initial model used (PDB code)	N/A	N/A	N/A	
Model resolution (Å)	3.7	3.2	6.5	
FSC threshold				
Model resolution range (Å)				
Map sharpening *B* factor (Å^2)				
Model composition				
Non-hydrogen atoms				
Protein residues				
Ligands				
B factors (Å^2)				
Protein				
Ligand				
R.m.s. deviations				
Bond lengths (Å)	0.006	0.010	0.004	
Bond angles (°)	0.668	0.890	0.833	
Validation				
MolProbity score	1.84	2.56	2.47	
Clashscore	12.78	17.42	26.94	
Poor rotamers (%)	0.32	3.51	0.19	
Ramachandran plot				
Favored (%)	96.53	93.99	89.90	
Allowed (%)	3.43	5.87	9.91	
Disallowed (%)	0.04	0.14	0.19	

Supplementary Discussion

Flagellins and other flagellar substrates, including FlgK, FlgL and FliD, are thought to be secreted N-terminal first via the type III secretion system due to the presence of N-terminal secretion signals[1, 2]. This is fully consistent with our observation that the N-terminal D0 helix of FliC folds first. However, this secretion mechanism requires a critical 180° turn in the polypeptide chain during assembly to achieve the final tail-to-tail orientation of flagellar axial proteins[3]. Based on our data, we propose that the N-terminus of the FliC polypeptide chain interacts with the D0-C domain of one FliD subunit as soon as it emerges in the cap cavity. These interactions anchor the polypeptide chain, while the rest of the chain is secreted upwards, where the 180° turn naturally occurs (Fig. 3d). Proper folding of the D0-N and D1-N helices of FliC continues to stabilize the flagellin monomer, with our data clearly showing that the D0-N and D1-N helices are refolded subsequently after the FliC chain is anchored (Fig. 3b). The D2-D3 domains of FliC are then pushed out of the cavity. Given the spatial patterns of domain appearance, we suggest that the D0-D1 domains of FliC are refolded within the FliD cavity at the incorporation site, while the D2-D3 domains are refolded outside the cap in a spontaneous manner.

Earlier studies demonstrated that the bacterial flagellar filament can assemble *in vitro* even in the absence of a cap complex; however, assembly is less efficient and requires a high concentration of purified (and potentially pre-folded) flagellin[4, 5]. Importantly, many studies, including our own, have firmly established that the cap plays an essential role in ensuring filament assembly *in vivo*[6-9]. We conclude that the cap facilitates the incorporation of flagellin subunits by providing a chaperone-like environment that guides the proper folding and self-assembly of flagellin monomers.

Interestingly, not only do flagellin molecules require a specialized cap structure for assembly, but so do other components such as the rod and hook, which are facilitated by the cap proteins FlgJ and FlgD, respectively [10-14]. The cap structures lack sequence similarity, and their incorporation mechanisms appear to differ significantly; however, there are notable similarities. Both structures display an asymmetric organization, with lower subunits featuring high-sitting leg domains that prime the next insertion sites[12]. Hence, for the hook cap FlgD, these terminal leg

domains likely play a crucial role, similar to the mechanism we propose for the function of the filament cap FliD. Therefore, both FlgD and FliD caps likely exhibit transient stabilization of assembly intermediates, ensuring proper hook or filament growth. The overall 3D structures of FlgD and FliD caps differ - for instance, terminal domains of FlgD form a helical bundle deeply embedded in the secretion channel, suggesting that the hook cap does not rotate[12]. This understanding is based on the structure of FlgD in its assembled state on top of the rod. Examining the hook cap's active structure during hook elongation might provide valuable insights and help determine whether a rotational mechanism is conserved across flagellar cap structures. However, the molecular mechanism of the rod cap FlgJ remains even less understood and requires detailed structural characterization. Notably, FlgJ not only facilitates rod assembly but also digests the peptidoglycan layer to enable penetration through the cell wall[10, 11]. In summary, our results reported highlight the diversity and adaptability in the strategies employed by different cap structures to facilitate flagella assembly.

Supplementary Discussion References

1. Chilcott G.S., Hughes K.T. (1998). The type III secretion determinants of the flagellar anti-transcription factor, FlgM, extend from the amino-terminus into the anti-σ28 domain. Mol. Microbiol. 30, 1029-1040. 10.1046/j.1365-2958.1998.01131.x.
2. Végh B.M., Gál P., Dobó J., Závodszky P., Vonderviszt F. (2006). Localization of the flagellum-specific secretion signal in *Salmonella* flagellin. Biochem. Biophys. Res. Commun. 345, 93-98. 10.1016/j.bbrc.2006.04.055.
3. Imada K. (2018). Bacterial flagellar axial structure and its construction. Biophys. Rev. 10, 559-570. 10.1007/s12551-017-0378-z 10.1007/s12551-017-0378-z.
4. Homma M., Iino T., Kutsukake K., Yamaguchi S. (1986). *In vitro* reconstitution of flagellar filaments onto hooks of filamentless mutants of *Salmonella typhimurium* by addition of hook-associated proteins. Proc. Natl. Acad. Sci. USA. 83, 6169-6173. 10.1073/pnas.83.16.6169.
5. Ikeda T., Asakura S., Kamiya R. (1985). "Cap" on the tip of *Salmonella* flagella. J. Mol. Biol. 184, 735-737. 10.1016/0022-2836(85)90317-1.
6. Al-Otaibi N.S., Taylor A.J., Farrell D.P., Tzokov S.B., DiMaio F., Kelly D.J., Bergeron J.R.C. (2020). The cryo-EM structure of the bacterial flagellum cap complex suggests a molecular mechanism for filament elongation. Nat. Commun. 11, 3210. 10.1038/s41467-020-16981-4.
7. Maki-Yonekura S., Yonekura K., Namba K. (2003). Domain movements of HAP2 in the cap–filament complex formation and growth process of the bacterial flagellum. Proc. Natl. Acad. Sci. USA. 100, 15528-15533. 10.1073/pnas.2534343100.
8. Postel S., Deredge D., Bonsor D.A. et al. (2016). Bacterial flagellar capping proteins adopt diverse oligomeric states. eLife. 5, e18857. 10.7554/eLife.18857.
9. Yonekura K., Maki S., Morgan D.G., DeRosier D.J., Vonderviszt F., Imada K., Namba K. (2000). The Bacterial Flagellar Cap as the Rotary Promoter of Flagellin Self-Assembly. Science. 290, 2148-2152. 10.1126/science.290.5499.2148.
10. Herlihey F.A., Moynihan P.J., Clarke A.J. (2014). The Essential Protein for Bacterial Flagella Formation FlgJ Functions as a Acetylglucosaminidase. J. Biol. Chem. 289, 31029-31042. 10.1074/jbc.M114.603944.
11. Hirano T., Minamino T., Macnab R.M. (2001). The role in flagellar rod assembly of the N-terminal domain of *Salmonella* FlgJ, a flagellum-specific muramidase. J. Mol. Biol. 312, 359-369. 10.1006/jmbi.2001.4963.
12. Johnson S., Furlong E.J., Deme J.C., Nord A.L., Caesar J.J.E., Chevance F.F.V., Berry R.M., Hughes K.T., Lea S.M. (2021). Molecular structure of the intact bacterial flagellar basal body. Nat. Microbiol. 6, 712-721. 10.1038/s41564-021-00895-y.
13. Matsunami H., Yoon Y.-H., Imada K., Namba K., Samatey F.A. (2021). Structure of the bacterial flagellar hook cap provides insights into a hook assembly mechanism. Commun. Biol. 4, 1291. 10.1038/s42003-021-02796-6.
14. Ohnishi K., Ohto Y., Aizawa S., Macnab R.M., Iino T. (1994). FlgD is a scaffolding protein needed for flagellar hook assembly in *Salmonella typhimurium*. J. Bacteriol. 176, 2272-2281. 10.1128/jb.176.8.2272-2281.1994.
