# Supplementary material for: The structure of the complete extracellular bacterial flagellum reveals the mechanism of flagellin incorporation
Source: Nat Microbiol. 2025 Jul 1;10(7):1741–57. doi: 10.1038/s41564-025-02037-0 (PMC12221982; doi:10.1038/s41564-025-02037-0)
Supplement: Supplementary file 2 — Reporting Summary [file 41564_2025_2037_MOESM2_ESM.pdf]

nature portfolio

Corresponding author(s): Julien Bergeron
Marc Erhardt

Last updated by author(s): 02/04/2025

Reporting Summary

Nature Portfolio wishes to improve the reproducibility of the work that we publish. This form provides structure for consistency and transparency in reporting. For further information on Nature Portfolio policies, see our Editorial Policies and the Editorial Policy Checklist.

Statistics

For all statistical analyses, confirm that the following items are present in the figure legend, table legend, main text, or Methods section.

n/a	Confirmed	
☐	☒	The exact sample size (*n*) for each experimental group/condition, given as a discrete number and unit of measurement
☐	☒	A statement on whether measurements were taken from distinct samples or whether the same sample was measured repeatedly
☒	☐	The statistical test(s) used AND whether they are one- or two-sided<br>*Only common tests should be described solely by name; describe more complex techniques in the Methods section.*
☒	☐	A description of all covariates tested
☒	☐	A description of any assumptions or corrections, such as tests of normality and adjustment for multiple comparisons
☐	☒	A full description of the statistical parameters including central tendency (e.g. means) or other basic estimates (e.g. regression coefficient) AND variation (e.g. standard deviation) or associated estimates of uncertainty (e.g. confidence intervals)
☐	☒	For null hypothesis testing, the test statistic (e.g. *F*, *t*, *r*) with confidence intervals, effect sizes, degrees of freedom and *P* value noted<br>*Give P values as exact values whenever suitable.*
☒	☐	For Bayesian analysis, information on the choice of priors and Markov chain Monte Carlo settings
☒	☐	For hierarchical and complex designs, identification of the appropriate level for tests and full reporting of outcomes
☒	☐	Estimates of effect sizes (e.g. Cohen's *d*, Pearson's *r*), indicating how they were calculated

Our web collection on statistics for biologists contains articles on many of the points above.

Software and code

Policy information about availability of computer code

Data collection	EPU 3 (Thermo Fisher) was used for cryo-EM data collection
Data analysis	Cryo-EM data was processed with CryoSPARC v4.3. Atomic models were generated with AlphaFold3, and the structures were refined with Phenix. Structural figures were generated with Pymol or ChimeraX.

For manuscripts utilizing custom algorithms or software that are central to the research but not yet described in published literature, software must be made available to editors and reviewers. We strongly encourage code deposition in a community repository (e.g. GitHub). See the Nature Portfolio guidelines for submitting code & software for further information.

Data

Policy information about availability of data

All manuscripts must include a data availability statement. This statement should provide the following information, where applicable:

- Accession codes, unique identifiers, or web links for publicly available datasets
- A description of any restrictions on data availability
- For clinical datasets or third party data, please ensure that the statement adheres to our policy

The Cryo-ET map of S. enterica flagellar tip has been deposited in the EMDB database. The coordinates and EM maps including the S. enterica cap-filament complex, the S. enterica HFJ, and the C. jejuni cap-HFJ complex, have been deposited in the PDB and EMDB databases with the following accession code: the cryo-ET map of

S. enterica flagellar tip, EMD-51555; the S. enterica cap-filament complex, PDB: 9GNZ, EMDB: EMD-51486; S. enterica HFJ, PDB: 9GO6, EMDB: EMD-51493; C. jejuni cap-HFJ complex, PDB: 9GSX, EMDB: EMD-51557.

Research involving human participants, their data, or biological material

Policy information about studies with human participants or human data. See also policy information about sex, gender (identity/presentation), and sexual orientation and race, ethnicity and racism.

Reporting on sex and gender	N.A
Reporting on race, ethnicity, or other socially relevant groupings	N/A
Population characteristics	N/A
Recruitment	N/A
Ethics oversight	N/A

Note that full information on the approval of the study protocol must also be provided in the manuscript.

Field-specific reporting

Please select the one below that is the best fit for your research. If you are not sure, read the appropriate sections before making your selection.

☒ Life sciences ☐ Behavioural & social sciences ☐ Ecological, evolutionary & environmental sciences

For a reference copy of the document with all sections, see nature.com/documents/nr-reporting-summary-flat.pdf

Life sciences study design

All studies must disclose on these points even when the disclosure is negative.

Sample size	Filament-cap complex: 15,225 particles<br>HFJ complex: 65,561 particles<br>HFJ-cap complex: 15,077 particles<br>These particles were obtained by rounds of 2D/3D classification, and were not pre-established prior to the experiment.
Data exclusions	Particles were excluded based on 2D and 3D classification, as described in the manuscript
Replication	The structures were obtained through averaging of all the particles for each of the complex. No biological replicates were performed.
Randomization	Resolution was estimated using the gold-standard FSC method (FSC=0.143), with the two half-maps generated by random particle distribution.
Blinding	Cryo-EM analysis relies on the averaging of 10,000 particles, and no blinding is required.

Reporting for specific materials, systems and methods

We require information from authors about some types of materials, experimental systems and methods used in many studies. Here, indicate whether each material, system or method listed is relevant to your study. If you are not sure if a list item applies to your research, read the appropriate section before selecting a response.

Materials & experimental systems

n/a	Involved in the study	
☐	☒	Antibodies
☒	☐	Eukaryotic cell lines
☒	☐	Palaeontology and archaeology
☒	☐	Animals and other organisms
☒	☐	Clinical data
☒	☐	Dual use research of concern
☒	☐	Plants

Methods

n/a	Involved in the study	
☒	☐	ChIP-seq
☒	☐	Flow cytometry
☒	☐	MRI-based neuroimaging

Antibodies

Antibodies used

(1) BD Difco™ Salmonellen H-Antiserum i. - Primary anti-FliC antibody (Cat # 11712894, lot # 1217577)
(2) Anti-FliD antibody - gift from Dr Tohru Minamino
(3) Anti-FlgK antibody - gift from Dr Tohru Minamino
(4) Secondary anti-rabbit antibody Alexa Fluor™ 488 (Invitrogen Cat # A-11034, lot # 1851447)
(5) Anti-DnaK antibody (Abcam Cat # ab69617, lot # 103701-2)
(6) Immun-Star Goat Anti-Mouse (GAM)-HRP Conjugate (BioRad Cat # 170-5047)
(7) Immun-Star Goat Anti-Rabbit (GAR)-HRP Conjugate (BioRad Cat # 170-5046)

Validation

(1) The commercially available Difco Salmonella H Antiserum is generally used in tube agglutination tests for the identification of Salmonella by flagellar (H) antigens and has been validated in our laboratory against Salmonella enterica serovar Typhimurium LT2 deletion mutants of FliC.
(2) Primary antibody was validated against Salmonella enterica serovar Typhimurium LT2 deletion mutants of FliD.
(3) Primary antibody was validated against Salmonella enterica serovar Typhimurium LT2 deletion mutants of FlgK.
(4) Immunofluorescence (IF), Flow Cytometry (Flow)
(5) WB, ICC/IF, ELISA; Reactivity tested on E. coli lysates, Primary antibody was validated in Salmonella enterica serovar Typhimurium LT2 in our laboratory and produces a single band of the expected size.
(6) Commonly used for WB
(7) Commonly used for WB

Plants

Seed stocks

Report on the source of all seed stocks or other plant material used. If applicable, state the seed stock centre and catalogue number. If plant specimens were collected from the field, describe the collection location, date and sampling procedures.

Novel plant genotypes

Describe the methods by which all novel plant genotypes were produced. This includes those generated by transgenic approaches, gene editing, chemical/radiation-based mutagenesis and hybridization. For transgenic lines, describe the transformation method, the number of independent lines analyzed and the generation upon which experiments were performed. For gene-edited lines, describe the editor used, the endogenous sequence targeted for editing, the targeting guide RNA sequence (if applicable) and how the editor was applied.

Authentication

Describe any authentication procedures for each seed stock used or novel genotype generated. Describe any experiments used to assess the effect of a mutation and, where applicable, how potential secondary effects (e.g. second site T-DNA insertions, mosiacism, off-target gene editing) were examined.
