# Supplementary material for: The structure of the complete extracellular bacterial flagellum reveals the mechanism of flagellin incorporation
Source: Nat Microbiol. 2025 Jul 1;10(7):1741–57. doi: 10.1038/s41564-025-02037-0 (PMC12221982; doi:10.1038/s41564-025-02037-0)
Supplement: Supplementary file 12 — Unprocessed western blots. [file 41564_2025_2037_MOESM12_ESM.pdf]

Unprocessed western blots

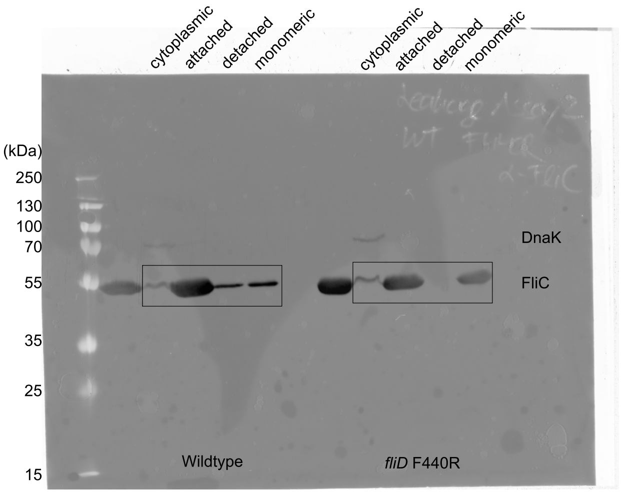

used in Figure 4h

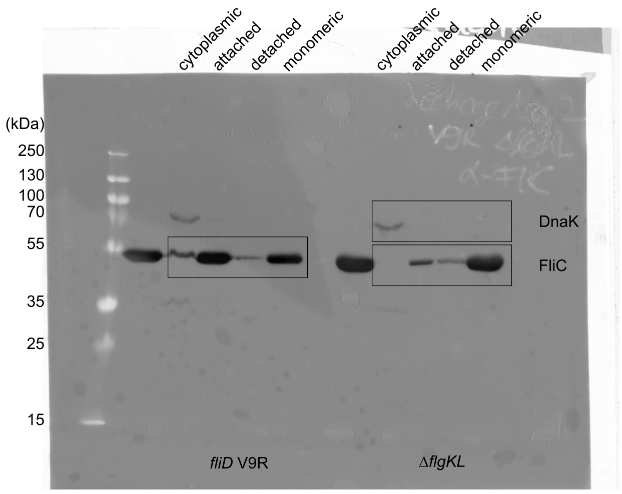

used in Figure 4h

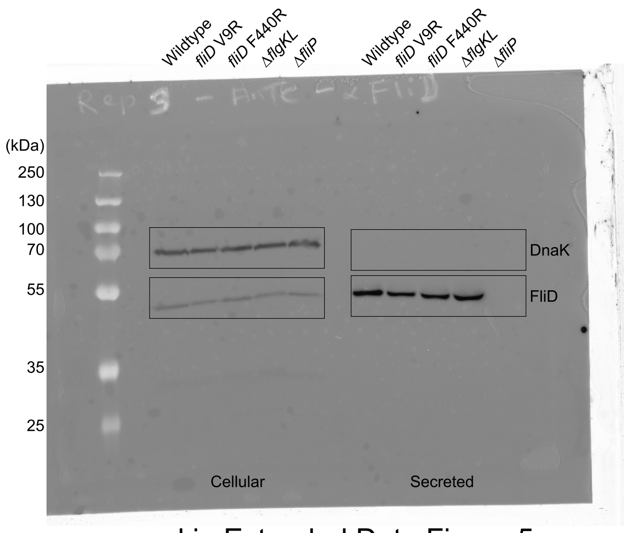

used in Extended Data Figure 5c

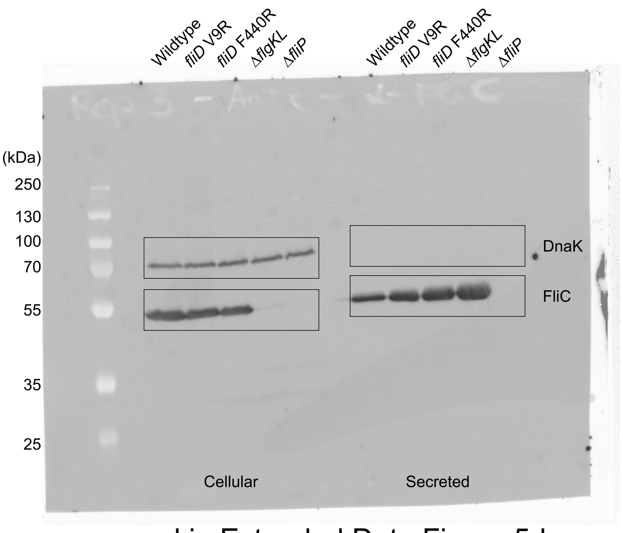

used in Extended Data Figure 5d

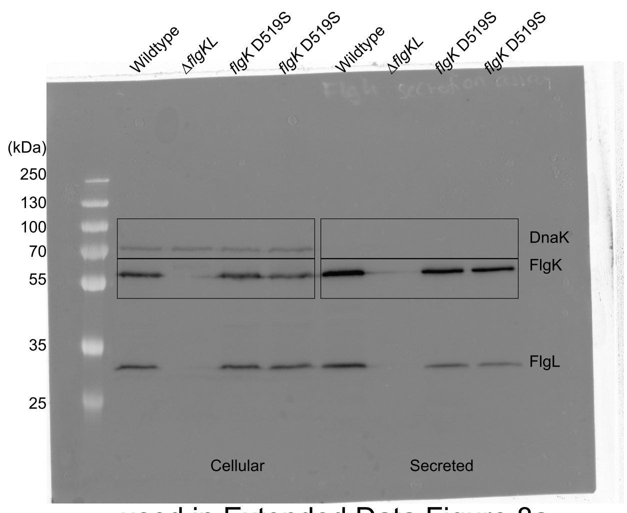

used in Extended Data Figure 8a
